# Emergence of Rare Species of Nontuberculous Mycobacteria as Potential Pathogens in Saudi Arabian Clinical Setting

**DOI:** 10.1371/journal.pntd.0005288

**Published:** 2017-01-11

**Authors:** Bright Varghese, Mushira Enani, Mohammed Shoukri, Sahar AlThawadi, Sameera AlJohani, Sahal Al- Hajoj

**Affiliations:** 1 Department of Infection and Immunity, King Faisal Specialist Hospital and Research Centre, Riyadh, Saudi Arabia; 2 Medical Specialties Department, King Fahad Medical City, Riyadh, Saudi Arabia; 3 National Biotechnology Centre, King Faisal Specialist Hospital and Research Centre, Riyadh, Saudi Arabia; 4 Department of Pathology and Laboratory Medicine, King Faisal Specialist Hospital and research Centre, Riyadh, Saudi Arabia; 5 Department of Pathology and Laboratory Medicine, King Abdulaziz Medical City, Riyadh, Saudi Arabia; University of Tennessee, UNITED STATES

## Abstract

**Background:**

Clinical relevance of nontuberculous mycobacteria (NTM) is increasing worldwide including in Saudi Arabia. A high species diversity of NTM’s has been noticed in a recent study. However, the identification in diagnostic laboratories is mostly limited to common species. The impact of NTM species diversity on clinical outcome is so far neglected in most of the clinical settings.

**Methodology/Principal Findings:**

During April 2014 to September 2015, a nationwide collection of suspected NTM clinical isolates with clinical and demographical data were carried out. Primary identification was performed by commercial line probe assays. Isolates identified up to *Mycobacterium species* level by line probe assays only were included and subjected to sequencing of *16S rRNA*, *rpoB*, *hsp65* and *16S-23S ITS region* genes. The sequence data were subjected to BLAST analysis in GenBank and Ez-Taxon databases. Male Saudi nationals were dominated in the study population and falling majorly into the 46–59 years age group. Pulmonary cases were 59.3% with a surprising clinical relevance of 75% based on American Thoracic Society guidelines. Among the 40.7% extra-pulmonary cases, 50% of them were skin infections. The identification revealed 16 species and all of them are reporting for the first time in Saudi Arabia. The major species obtained were *Mycobacterium monascence* (18.5%), *M*. *cosmeticum* (11.1%), *M*. *kubicae* (11.1%), *M*. *duvalli* (7.4%), *M*.*terrae* (7.4%) and *M*. *triplex* (7.4%). This is the first report on clinical relevance of *M*. *kubicae*, *M*. *tusciae*, *M*.*yongonense*, *M*. *arupense* and *M*.*iranicum* causing pulmonary disease and *M*. *monascence*, *M*. *duvalli*, *M*. *perigrinum*, *M*. *insubricum*, *M*. *holsaticum* and *M*. *kyorinense* causing various extra-pulmonary diseases in Saudi Arabia. Ascites caused by *M*. *monascence* and cecum infection by *M*. *holsaticum* were the rarest incidents.

**Conclusions/Significance:**

To the first time in the country, clinical significance of various rare NTM’s are well explored and the finding warrants a new threat to the Saudi Arabian clinical settings.

## Author Summary

Nontuberculous mycobacteria (NTM) are ubiquitous in nature and they are opportunistic pathogens. In the last decade, infections caused by NTM’s increased—around the world in immune-suppressed and immune-competent individuals and Saudi Arabia is not an exception. Developments in diagnostic technologies increased the identification of several new or rare species of NTM’s. Indeed, the species diversity of NTM has a direct impact on clinical outcome and therapies. Saudi Arabian clinics so far report only the common species of NTM’s and rare species are mostly neglected due to the lack of proper infrastructure or ignorance. To the first time in the country, an exploration on the existence of clinically relevant rare NTM species was conducted on a nationwide level. The findings showed a huge diversity of rare NTM species causing both pulmonary and extra-pulmonary diseases. Clinical relevance of pulmonary infection based on American Thoracic Society guidelines was confirmed as an aggressive 75%, which is really alarming. Interestingly, 16 species of NTM’s were isolated in the study, and all of them are reporting for the first time in country. Overall finding shows Saudi Arabia faces serious threat from rare NTM species with high clinical significance and warrants the immediate need for more advanced infrastructure.

## Introduction

In the last decade, the prevalence of pulmonary and extra-pulmonary diseases caused by nontuberculous mycobacteria (NTM) has been increased [1–7]. This elevation in case rates, whether it is a real emergence or due to the development of advanced diagnostic tools is still unclear. On the other hand, the elevation in immunosuppressive conditions including infectious or non-infectious diseases and therapies contribute considerably in this phenomenon. To date, more than 140 species of NTM’s have been described from different sources with varying pathogenicity and almost 50 species were identified in the last 8 years alone [8]. However, in literature only a small number of reports are available about the new species as their role in clinical microbiology is largely undetermined. Mostly, the species defined as “rare” will remain unrecognized or misidentified due to the lack of proper resources, lack of knowledge or ignorance [8]. The clinical characteristics of diseases caused by the rare or new NTM’s are still not fully established. The advancement in technologies such as genome sequencing, line probe assays, high performance liquid chromatography (HPLC) and matrix assisted laser desorption ionization time-of-flight (MALDI-TOF) to identify the NTM species increased the detection of rare and new NTM species. However, accessibility to such tools in resource poor settings is a major concern for timely identification. Thus, the species level identification is mostly neglected regardless of its importance in clinical outcome.

Following the global trend of NTM prevalence, Saudi Arabia also reports with an increasing numbers of NTM diseases [7, 9]. In 2013, Varghese et al. reported in the first national level study a highly diverse population of clinically relevant NTM’s with the potential of causing pulmonary and extra-pulmonary diseases [9]. Interestingly, a new species of pathogenic mycobacteria also has been identified from the country named *M*. *riyadhense* [10]. However, the diagnosis of NTM’s is mostly limited to the common species only in majority of the laboratories, because of the lack of infrastructure. Therefore, there is no data available on the existence of rare NTM species in the country so far.

To explore the diversity of rare NTM species with clinical relevance in the Saudi Arabian clinical setting, a prospective analysis on a nationwide isolate collection has been designed. Sequencing of *16S rRNA*, *rpoB*, *hsp65* and *16S-23S ITS* region genes were carried out to identify the species. Clinical significance of pulmonary isolates in the study was determined by applying the criteria based on American Thoracic Society (ATS) guidelines for NTM pulmonary diseases [11]. The species diversity and clinical significance of each isolates have been evaluated.

## Materials and Methods

### Study population

This study has been conducted as part of the first national NTM surveillance survey of Saudi Arabia. The duration of collection was 18 months, from April 2014 to September 2015. All the suspected NTM isolates from different mycobacterial diagnostic laboratories were collected and transferred to the Mycobacteriology Research Section of King Faisal Specialist Hospital and Research Centre, Riyadh. The demographical and clinical data were collected by using standard data collection forms without keeping any patients identifiers. Pulmonary cases were defined as clinically relevant based on ATS guidelines [11]. Briefly, a minimum of two positive cultures from separate sputum samples or at least one positive culture from bronchial wash, lavage or one positive culture from trans-bronchial or other lung biopsy showing mycobacterial- histopathological features were considered as clinically relevant to define NTM pulmonary disease.

### Primary identification and enrollment

Isolates were maintained on Lowenstein Jensen slants and modified Middle Brook 7H9 medium (Becton Dickinson, USA). The genomic DNA was extracted by using the QIAamp DNA Mini kit (Qiagen, Germany). The primary screening to identify the NTM’s was carried out by commercially available line probe assay kit- Genotype MTBC (Hain Life science, Germany). The non-MTBC isolates were initially identified with Genotype Mycobacterium CM kit (Hain Life science, Nehren, Germany) and unidentified isolates were further tested with Genotype Mycobacterium AS kit (Hain Lifescience, Nehren Germany).

Isolates which were detected by the Genotype Mycobacterium AS assay up to genus level (*Mycobacterium species*) only were included in the study as “unidentified” species. This study has been reviewed and approved by the Office of Research Affairs in King Faisal Specialist Hospital and Research Centre, Riyadh, Saudi Arabia.

### Sequencing analysis

Sequencing assay was carried out by using the BigDye Terminator cycle sequencing chemistry kits (Applied Biosystems, CA, USA) in DNA analyzer 3730 (Applied Biosystems, CA, USA). The first attempt of identification was based on a 645-655bp hyper variable region of the *16S rRNA* and a 342bp region of *rpoB* genes based on previously standardized protocol [12, 13]. Isolates which could not be identified by *16S rRNA* and *rpoB* gene sequencing were subjected to further sequencing of highly conservative regions of two more genes *hsp65* (439bp) and *16S-23S ITS region* (480bp) according to previously validated primers [14–16].

### Data analysis

The line probe assay test strips were scanned with Genoscan (Hain Lifescience, Germany) and the results were interpreted with the Blotrix software (Hain Lifescience, Germany). The sequence base calling and assembly were carried out by using Sequence Analysis software v5.3.1 (Applied Biosystems, USA) and Lasergene core suite 12 (DNA STAR, WI, USA) respectively. Assembled sequences were subjected to BLAST analysis in NCBI GenBank and EzTaxon (http://www.ezbiocloud.net/identify; 1*6S rRNA* Based Database) online data bases [17]. A stringent similarity index of ≥99–100% was kept with the type strain in GenBank and EzTaxon. Statistical data analysis was carried out by using SPSS V20.0 software package (IBM, USA).

## Results

### Demographical and clinical summary

During the study period, 510 suspected NTM clinical isolates were collected and subjected to line probe assay identification. Of the total, 27 isolates met the inclusion criteria and enrolled for further analysis. Demographical summary of the study subjects showed 22 (81.5%) of the enrolled cases were Saudi nationals with a male (77.8%) gender domination. Interestingly, the age group of the study subjects showed a predominance of 46–59 years (48.2%). Seven cases out of 27 had a previous history of tuberculosis and 3 were reactive to HIV antigens. The major comorbidities noticed among the study subjects were rheumatoid arthritis (18.5%), malignancies (18.5%) and diabetes (14.8%). Considerable percentage of Chronic Obstructive Pulmonary Disease (COPD), asthma and bronchiectasis also were observed (Table 1).

**Table 1:** Study summary of 27 rare NTM isolates from Saudi Arabia.

**Isolate**	**Species**	^1^**Type Strain/** *16S rRNA* **Similarity %**	^2^**Type Strain/** *rpoB* **Similarity %**	**Age/Gender**	**Nationality**	**Specimen type**^3^	**Clinical relevance**^4^	**Treatment**^5^	**Underlying Conditions**^6^
**1.**	*M*.*arupense*	NR_043588/99.6	JN571215/99.0	30/F	Saudi	BAL^**2**^	Yes	RIF, EMB, CLR	HL,COPD,HIV
**2.**	*M*.*cosmeticum*	NR_025787/ 99.8	AY262742/ 96.8	39/M	Saudi	sputum^**1**^	No	-	AS,PTB,HIV
**3.**	*M*.*cosmeticum*	NR_025787/ 99.0	AY262742/ 96.0	48/M	Saudi	sputum^7^	Yes	-	Lung Transplant
**4.**	*M*.*cosmeticum*	NR_025787/ 99.2	AY262742/ 96.6	59/M	Yemen	skin^3^		-	DM
**5.**	*M*.*duvalii*	NR_026073/99.1	AY544907/99.1	56/F	Saudi	sputum^**1**^	No	-	NHL,RA
**6.**	*M*.*duvalii*	NR_026073/99.7	AY544907/99.3	63/M	Saudi	peritoneum^**1**^	-	CF,AK	HL,CAPD
**7.**	*M*.*holsaticum*	NR_028945/99.4	AY859705/98.6	47/M	Saudi	cecum^**2**^	-	EMB,OF	DM,UC,RA
**8.**	*M*.*insubricum*	NR_125525/99.3	EU605693/90.0	52/M	Eritrea	skin^**1**^	-	-	PTB
**9.**	*M*.*iranicum*	KU861842/99.6	JQ90669809/98.9	51/M	Saudi	BAL^**2**^	Yes	-	PTB
**10.**	*M*.*kubicae*	HM022200/99.3		69/F	Saudi	BAL^**1**^	Yes		RA
**11.**	*M*.*kubicae*	HM022200/99.7		49/M	Saudi	sputum^**1**^	No	-	PTB
**12.**	*M*.*kubicae*	NR_025000/99.1		51/F	Saudi	sputum^3^	Yes	-	DM,CVD,RA
**13.**	*M*.*kyorinense*	NR_041663/98.7	JQ717032/99.2	14/M	Saudi	lymphnode^**1**^		EMB, CLR	HL
**14.**	*M*.*monascence*	NR_041723/99.5	HM229793/99.	68/M	Saudi	sputum^6^	Yes	-	PTB,AS
**15.**	*M*.*monascence*	NR_041723/99.3	KU361327/99.0	52/M	Saudi	sputum^**2**^	Yes	-	COPD, BE
**16.**	*M*.*monascence*	NR_041723/99.0	HM229793/99.0	2/F	Saudi	BAL^**1**^	Yes	-	COPD
**17.**	*M*.*monascence*	NR_041723/99.6	KU361327/99.6	52/M	Saudi	Ascitic fluid		-	COPD, IBD
**18.**	*M*.*monascence*	NR_041723/99.4	HM229793/98.4	63/M	Saudi	Lymphnode		-	BE
**19.**	*M*.*novocastrense*	NR_029208/99.2	AY859704/98.9	74/M	Saudi	sputum^**2**^	Yes	-	BE,RA
**20.**	*M*.*perigrinum*	NR_114447/99.1	AY147166/98.7	48/M	Sudan	skin^**1**^	-	-	PTB
**21.**	*M*.*marinum*	NR_113366/ 99.2	CP000854/ 98.2	33/M	Sudan	skin^**1**^		RIF,CLR	-
**22.**	*M*.*terrae*	NR_117886/99.0	JN571264/98.9	68/M	Saudi	sputum^**1**^	No	AK,CF	COPD, DM
**23.**	*M*.*terrae*	NR_115677/99.1	AY544967/99.0	49/M	Nigeria	skin		-	PTB,AS
**24.**	*M*.*triplex*	NR_117226/99.1	AY544970/99.0	69/M	Saudi	BAL	Yes	-	CVD,AS
**25.**	*M*.*triplex*	NR_117226/99.5	KF856622/98.7	48/F	Saudi	lymphnode		AK, EMB,CLR	HIV
**26.**	*M*.*tusciae*	NR_117885/99.3		64/M	Saudi	sputum^3^	Yes	INH, EMB,RIF	BE, HCA
**27.**	*M*.*yongonense*	KF_224994/99.8	JF271829/99.1	16/M	Saudi	BAL^**2**^	Yes	-	COPD,NHL

*1 16S rRNA* gene closest match- Gene bank accession number.

2 *rpoB* gene closest match- Gene bank accession number.

3 Number shows how many times the species was isolated from the patient.

4 ATS and IDSA NTM pulmonary disease criteria met.

5 Drugs (on use while collecting isolates)- EMB- Ethambutol; CLR- Clarithromycin; AK-Amikacin; RIF-Rifampicin; CF-Ciprofloxacin, OF-Ofloxacin.

6 HL-Hodgkin Lymphoma; DM- Diabetes Mellitus, NHL- Non-Hodgkins Lymphoma; UC-Ulcerative Colitis; RA- Rheumatoid Arthritis; PTB- Previous Tuberculosis; BE- Bronchiectasis; COPD- Chronic Obstructive Pulmonary Disease; IBD- Inflammatory Bowel Disease; HCA- Hepatocellular Carcinoma; AS- Asthma; CAPD- Continuous Ambulatory Peritoneal Dialysis, CVD- Cardio Vascular Diseases, INH-Isoniazid.

### Diversity of NTM species

**Fig 1:**
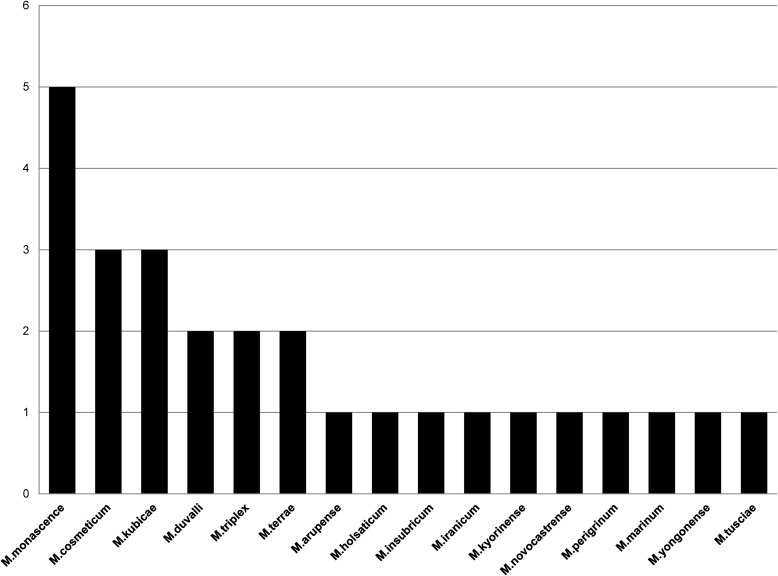
Species diversity of rare NTM’s isolated in Saudi Arabia. The figure shows the overall diversity of 27 rare NTM isolates from pulmonary and extra-pulmonary samples.

Analysis of *16S rRNA*, *rpoB*, *hsp65 and 16-23S ITS region* genes, showed an extreme diversity of NTM species distributed to 16 species. The majorly detected species were *M*. *monascence* (18.5%), *M*. *cosmeticum* (11.1%), *M*.*kubicae* (11.1%), *M*. *duvalii* (7.4%), *M*. *triplex* (7.4%) and *M*. *terrae* (7.4%). Rest of the 10 species were reported with one case each (3.7%) (Fig 1).

### Site of infection and clinical relevance

The major site of infection observed in the study was pulmonary (59.3%). Clinical relevance of pulmonary isolates based on the ATS guidelines was dominating (75%). Of the 16 pulmonary cases, four cases did not qualify the clinical relevance guidelines and thus considered as colonization. Clinically relevant diseases were caused *by M*. *arupense*, *M*. *cosmeticum*, *M*. *iranicum*, *M*. *kubicae*, *M*. *monascence*, *M*. *novocatrense*, *M*. *tusciae* and *M*. *yongonense* (Table 1).

On the other hand, extra-pulmonary involvement was found with 11 (40.7%) cases. Among extra-pulmonary cases, skin (45.5%) was the most affected site of infection followed by lymphnode (27.3%). *M*. *insubricum*, *M*. *perigrinum* and *M*. *marinum* were found exclusively causing granuloma or sepsis. Interestingly, all these five skin infections were observed among non-Saudi patients. Cecum infection by *M*. *holsaticum* and ascites caused by *M*. *monascence* were the rarest incidents in this study (Table 1).

## Discussion

For the first time in Saudi Arabia, the existence of rare NTM species has been explored on a nationwide collection of clinical isolates. The findings showed a strong presence of clinically relevant NTM rare species in the Saudi Arabian clinical settings. The species diversity of rare NTM’s causing both pulmonary and extra-pulmonary diseases was huge (16 species). To date, all of these 16 species with clinical relevance are reporting for the first time in the country.

Demographical analysis showed predominance of male Saudi nationals and mainly the age group 46–59 years and a similar domination had been noticed in a recent nationwide study of NTM prevalence [9]. Clinical data showed various comorbidities existed among the study group. Rheumatoid arthritis and various malignancies were the major problems followed by diabetes. There were no studies so far analyzed the reasons behind the predominance of male Saudi nationals towards the NTM disease. Perhaps, there were some speculations like higher rate of consanguinity existed in the community which leads to several genetic susceptibility diseases, increasing rate of immunosuppressive therapies and various malignancies in the geographical region [18–20]. Indeed, the confounding factors for this predominance need further detailed scientific exploration.

In this study, pulmonary diseases caused by rare NTM species were predominant with higher clinical relevance (75%). Of the 16 identified species, 11 species except *M*. *insubricum*, *M*. *kyorinense*, *M*. *holsaticum*, *M*. *perigrinum* and *M*. *marinum* were isolated from respiratory samples. Of the 11 species, except *M*. *duvalii* and *M*. *terrae* all the others reported with clinically relevant diseases. Moreover, most of these species are isolated very rarely from clinical specimens with relevance so far around the world [21]. Interestingly, to date, *M*. *tusciae*, *M*. *yongonense*, *M*. *novocastrens* and *M*. *monascence* pulmonary diseases were reported in hardly 2–3 publications. Therefore, these findings are important as it shows and confirms the growing problems with rare NTM species in Saudi Arabia as well for the first time in the Gulf Cooperation Council (GCC) states also [22] [21, 23, 24]. The higher clinical relevance established after consulting the ATS guidelines shows the increasing potential of NTM respiratory diseases in the country. Supportively, NTM respiratory diseases caused by various species have been observed in a recent nationwide study in the country [9]. In the current study majority of the isolation was from sputum samples, except six bronchial washes. The frequency of isolation from sputum was peaked up to 7 occasions for a case of *M*. *cosmeticum*. The higher frequency shows the increasing potential of NTM’s as en establishing pathogen in the clinical settings. Most of the NTM species isolated in the current study causing pulmonary diseases are not only rare in Saudi Arabia but also around the globe. In concordance to the current findings, previous global studies showed an increasing prevalence of NTM’s and particularly the domination of pulmonary isolates. The existence of numerous rare or new species was observed in most of the large level studies [5–7, 21]. The increased clinical relevance is a warranting key to be vigilant on the pathogenicity and potential of NTM’s to cause confirmed diseases rather than colonization.

In the current study, 11 cases of extra-pulmonary diseases were observed with a predominance of skin infections (50%) caused by *M*. *cosmeticum*, *M*. *insubricum*, *M*. *perigrinum*, *M*. *marinum* and *M*. *terrae*. The *M*. *perigrinum* and *M*. *marinum* were isolated from two immigrant patients from the Western coast of the country, where the major fishing harbors located with considerable number of foreign fishermen. The skin infection caused by *M*. *marinum* and *M*. *perigrinum* among people who work in fisheries or swimming pool maintenance is generally reported elsewhere [25, 26]. The rarest cases in the study were the ascites caused by *M*. *monascence* and cecum infection caused by *M*. *holsaticum*. To our knowledge, this might be the first cases of the same reporting globally. On the other hand, lymphadenitis caused by *M*. *kyorinense* and *M*. *triplex* are rare manifestations which have been reported in only two or three cases so far globally and the current study also found a case of each [27, 28]. The infections caused by *M*. *duvalii* and *M*. *monascence* in patients undergoing peritoneal dialysis are reported for the first time in Saudi Arabia and in GCC nations. Such cases are rarely reported around the world [8].

## Conclusion

As the first report on the existence of rare NTM species in Saudi Arabia, the findings showed an alarming diversity of clinically relevant NTM’s causing both pulmonary and extra-pulmonary diseases. The diagnostic facilities in the country requires more advanced infrastructure to identify the rare species on time, as it influence the final clinical outcome and treatment adversely.

### Sequence deposit accession numbers

The following *16S rRNA* gene sequences have been deposited in GenBank/DDBJ/EMBL data bases. *M*. *monacense* (KY287007), *M*. *iranicum* (KY287008), *M*. *kubicae* (KY287009), *M*. *cosmeticum* (KY287010), *M*. *duvalii* (KY287011), *M*. *terrae* (KY287012), *M*. *arupense* (KY287013) and *M*. *novocastrense* (KY287014).
